# Innovation as a practice: Why automation will not kill innovation

**DOI:** 10.3389/fpsyg.2022.1045508

**Published:** 2023-01-06

**Authors:** Dulce M. Redín, Goretti Cabaleiro-Cerviño, Ignacio Rodriguez-Carreño, German Scalzo

**Affiliations:** ^1^Department of Business, Uinversity of Navarra, School of Economics and Business, Pamplona, Spain; ^2^Business Ethics Department, Universidad Panamericana, School of Economics and Business, Mexico City, Mexico

**Keywords:** AI, tradition, virtues, innovation, automation, MacIntyre

## Abstract

As a result of contemporary culture’s focus on continuous innovation and “change before you have to,” innovation has been identified with economic gains rather than with creating added value for society. At the same time, given current trends related to the automation of business models, workers seem all but destined to be replaced by machines in the labor market. In this context, we attempt to explore whether robots and Artificial Intelligence (AI) will be able to innovate, and the extent to which said activity is exclusively inherent to human nature. Following the need for a more anthropological view of innovation, we make use of MacIntyrean categories to present innovation as a domain-relative practice with creativity and practical wisdom as its corresponding virtues. We explain why innovation can only be understood within a tradition as it implies participating in inquiry about the principle and end of practical life. We conclude that machines and “intelligent” devices do not have the capacity to innovate and they never will. They may replicate the human capacity for creativity, but they squarely lack the necessary conditions to be a locus of virtue or engage with a tradition.

## Introduction

The current situation calls on us to rethink innovation, its role in history and in our daily lives. Within a context of increasing global competition, an accelerating pace of change, increasing complexity and uncertainty, the scarcity of resources, and increases in prices, companies need to continually update their knowledge. Many companies have placed considerable emphasis on innovation activities as a way to adapt their production methods, processes, and systems to the current challenging context. At the same time, AI has been adopted within a broad range of organizational settings, transforming a variety of common workplace tasks. For example, recruitment processes now routinely use facial recognition to screen candidates (Van den Broek et al., 2021), sales functions have become automated (Pachidi et al., 2021), and new forms of employee surveillance are being deployed, often with harmful consequences, to optimize labor (Rahman, 2021). AI not only represents a way to achieve cost and productivity benefits, but also presents itself as a fundamental innovation upon the tools through which we innovate (Cockburn et al., 2019). Yet, sometimes the outcomes of innovation seem to be disconnected from and poorly oriented toward true prosperity (Martinez-Echeverría and Scalzo, 2015), and the race for innovation and automation of work has exacerbated a division of labor characterized by precarious work, automatic management, and deskilling (Cherry, 2016).

Since Schumpeter (1934) identified innovation as a key source of economic growth and development, several studies have demonstrated the positive impact of innovation on firm performance (Choi et al., 2012; Psomas et al., 2018), on economic growth (Hölzl and Friesenbichler, 2010; Santi and Santoleri, 2017), on success when entering a new market (Dosi, 1988), on increasing existing market share, on positive reputation building among customers, and on the capacity to overcome problems and better adapt to new environments (Teece et al., 1997; Al Kurdi et al., 2020; Kurdi et al., 2020; Hayajnedh et al., 2021). Many see it as a crucial factor for cultivating sustainable competitive advantage (Barney, 1991; Becheikh et al., 2006). Moreover, innovation is well considered within society, and organizations are expected to anticipate and keep pace with change, creating a culture of continuous innovation. Jack Welch, the legendary CEO of GE, used to exhort his audience to “Change before you have to.”

At the same time, the majority of large firms listed on the stock market are currently seeking to automate their business model in order to grow, to be more efficient, and to please investors, who seem to value such changes positively (MarketWatch, 2018). These automation technologies include robotic process automation (RPA), intelligent automation, and AI-based decision-making tools, among others. The Mckinsey Global Institute (2017) Report states that automation in the labor market will bring unprecedented consequences. Laura Tyson, Chief Economic Advisor to former US President William Clinton and a professor at the University of California at Berkeley, argues, “This is the first time we see that technology could reduce the demand for human workers” (Rotman, 2018).

Discussion around the effects of automation has come to the fore in recent years, with particular emphasis on its impact on the labor market (Bakhshi et al., 2017). Yet, the side effects of this phenomenon have barely been addressed in the literature and, to our knowledge, nothing is being said about the future of innovation itself. This paper attempts to fill this gap.

Some of the studies regarding the effect of automation on the labor market underline the following predictions: (i) Frey and Osborne (2013, 2017) conclude that 47% of jobs in the United States are susceptible to being automated. (ii) The results of the Mckinsey Global Institute (2017) estimate that 51% of US jobs will be automated by 2030; 15% of the new cars sold in 2030 will be fully automated; cities will become intelligent; and our future everyday lives will be different due to the predominance of the “Internet of Things.” (iii) Bughin and Van Zeebroack (2017) predict that proven AI technologies have the potential to replace up to half of all work activities carried out by humans and show that 60% of all occupations consist of approximately 30% automatable activities. (iv) Nedelkoska and Quintini (2018) conclude that, in 32 countries around the world, people working in the manufacturing industry and the agricultural sector are at high risk of being replaced due to automation and that women, workers with less education and those who work under a learning contract are more likely to have their jobs computerized. (v) Finally, a study by PricewaterhouseCoopers (2018) concludes that administrative or office workers face the greatest potential impact in the short and medium term, with automation of their work reaching 49% by the end of the 2020s (Hawksworth et al., 2018).

If workers are destined to be replaced by machines in the labor market, what happens to innovation? Who is going to innovate? This paper attempts to explore whether current automation technologies and Artificial Intelligence (AI) will be able to innovate, and the extent to which this activity is inherent to human nature. In order to do so, we use MacIntyrean practice-institution categories to understand the nature of innovation and its connection with the idea of tradition. Virtue Ethicists agree that the social philosopher Alasdair MacIntyre has provided a great impetus to virtue ethics tradition applied to business and economic activities (Moore and Beadle, 2006; Ferrero and Sison, 2014). Even though he was rather critical to business, his moral renewal project “After Virtue” [which includes MacIntyre (1988, 2007, 1990, 1999)] became a key milestone in the rehabilitation of virtue ethics. In particular, he criticizes liberal philosophy and the culture of individualism, providing a conceptual framework that highlights the importance of communities and tradition in the quest of practical wisdom and the common good (MacIntyre, 2016: 92). Some scholars (Wicks, 1997; Hager, 2011) have criticized his proposal, for being unrealistic and idealistic, far from the reality of modern markets and economic systems. However, the fact is that MacIntyre “practice-institution” distinction has been a key theoretical contribution for understanding modern corporations (Moore, 2002; Moore and Beadle, 2006) and the relationships between work and technology (Pinto-Garay et al., 2022b). In this paper, making use of MacIntyrean categories, we aim to describe the main features of innovation focusing on its social embeddedness.

The following provides an overview of this paper’s argument and layout. Section 2 explains what innovation is (its main types and characteristics). Section 3 frames innovation within the MacIntyrean category of practice and connects it with the traditions of rational inquiry. Innovation is a domain-relative practice, with creativity and practical wisdom as its corresponding virtues. Some authors (Steen, 2013; Astola et al., 2021) have emphasized the value of creativity as a virtue possessed by collectives, rather than individuals, in relation to collective innovation (Hill, 2014). We follow an individual account of virtue and we make use of MacIntyrean integrative categories of practice, biography, and tradition to explain the collective and cooperative dimensions of innovation. Section 4 reviews the efforts underway to achieve the necessary capacities for innovation and creativity through AI and automation technologies. Section 5 analyzes whether innovation and creativity are specifically human traits and to what extent they can be carried out by machines and robots. We conclude that innovation is a (domain-relative) practice that is specifically human, as it entails a strong connection with virtues and can only be understood within the social embeddedness found in communities and traditions. Innovation has a teleological component and implies prudential actions that demand rational knowledge and free will to act voluntarily. In its current state of development, AI does not exhibit these features.

## A review of the contemporary concept of innovation

The term innovation comes from the Latin *innovare*, which means to change or alter things by introducing novelty. Broadly speaking, from the point of view of economics, innovation can be considered anything that contributes to increasing the value that exists in society, that is, to improving the welfare of those who live in it.

One of the problems that plagues innovation management corresponds to the variation in what people understand for the term, which they often confuse with invention. Innovation has been classified in many different ways in the literature, each of which has focused on different outcomes. For example, Schumpeter (1934) classified innovations depending on the object modified, namely product, process, market, or organization; Knight (1967) distinguished between innovations in organizational structure, the production process, people, and product or service; Trott (2005) classified different innovation efforts into innovations in marketing, product, production, management, organizational, service and process; and, Francis and Bessant (2005) distinguished between innovations in process, position, paradigm, and product. In this context, the OECD’s *Oslo Manual* provided a definition geared toward consensus. There, innovation is defined as: *“The introduction of a new or significantly improved product (goods or services), process, new marketing method or new organizational method in the company’s internal practices, workplace organization or external relations”* (OECD, 2005: 46). Furthermore, the OECD also provides a firm-level definition of innovation that refers to planned changes in a firm’s activities aimed at improving the firm’s performance.

In fact, the core of the innovation process includes two main stages involving the creation, recognition, or discovery of innovative ideas, opportunities, and solutions and the subsequent development or exploitation of these ideas to evaluate them and select one or more of them (e.g., Kijkuit and van den Ende, 2007). Those two steps require creativity and out-of-the-box-thinking (Shane, 2003; Martin and Wilson, 2016). Therefore, innovation activities should be strategically designed in advance in accordance with the firm’s specific objectives while invention is only the first step in a process of bringing a good idea to widespread and effective use. Long ago, in 1934, Schumpeter rightly pointed out that the discovery and execution of an innovation are two entirely different things: “The pure new idea is not adequate by itself to lead to implementation … It must be taken up by a strong character (entrepreneur) and implemented through his influence” (Śledzik, 2013: 91). For example, data from a Study by the Product Development and Management Association (2012) show that it takes about 3,000 raw ideas to produce one significantly new and successful product (Markham and Lee, 2012), thus underscoring the difficulty in properly managing the innovation process.

Generally speaking, innovation sources can be divided into internal and external. Internal sources refer to the R&D activities completed by existing departments, their innovative practices, educational events, as well as initiatives from employees that generate information and knowledge in firms. Over time, this internal base of knowledge is enhanced with internal learning on the part of employees. Previous research has shown that internal R&D activities transform information into knowledge, improve employees’ learning capacity and enhance their ability to absorb external knowledge (Cohen and Levinthal, 1989, 1990; Griliches, 1995). The Resource Based View (Barney, 1991) states that these internal resources alone determine the firm’s performance and, in turn, its ability to generate sustainable competitive advantage. However, firms nowadays cannot solely rely on internal sources of knowledge and increasingly must complement internal knowledge with external sources in order to innovate. In fact, at this point, the folklore surrounding the “sole innovator” has disappeared and experts have accepted that firms cannot innovate in isolation (De Bresson and Amesse, 1991; Bidault and Cummings, 1994). In this context, the concept of dynamic capabilities defines innovation not just as the result of good management of internal resources, but also as a social process of adaptation to rapid changes in the environment (Teece et al., 1997).

To this end, in the literature, the concept of networks has been increasingly used to analyze sources of external knowledge. In that sense, the network theories of innovation have developed from more restricted networks with clients, suppliers, and research partners to consider a broader range of institutional and social actors as sources of information, known as systems of innovation (Lundvall, 1992, 1995; Nelson, 1993; Edquist, 1997; Edquist and Hommen, 1999). The basic network approach is mainly focused on firm-to-firm relationships; meanwhile, the extended systems of an innovation approach take into account complex interactions between a broader range of actors such as firms, universities, research institutes, educational organizations, financial institutions, public support organizations, etc. within a diverse economic, institutional, social, political, cultural, and geographical context. There is greater focus on the importance of the relations between companies and other organizations for innovating and analyzing the interplay of a firm’s internal operations and external relations (Tödtling et al., 2009). In a broad sense, innovation systems assume that information is immersed in networks and is the result of relationships within the network (Acs, 2000), and that these networks increase the firm’s ability to identify and generate innovation opportunities (Powell, 1990; Burt, 2000). It foresees that the more constant and intense these relationships are, the more likely it is that the information will be used to develop radically new innovations (Amara and Landry, 2005).

In order to generate knowledge-based innovations, companies must implement technological as well as relational tools (Lengrand and Chatrie, 1999). Technological tools involve the acquisition and use of information technologies that are also available to other companies; thus, they cannot be responsible for generating sustainable competitive advantages. Instead, relational tools are behind the creation of valuable competitive advantages. This has been named “social capital” and refers to the different ways of doing business and interacting within the network.

Even though social capital can take many forms, in the context of innovation, it essentially refers to trust and network (Fountain, 1998; Dasgupta and Serageldin, 2000; Lesser, 2000). On the one hand, trust is generated over time with interactions and is related with a high probability to innovate. On the other hand, networks refer to the ability to create reliable and effective communication channels inside and outside the company (Le Bas et al., 1998). Previous research has shown that social capital reduces transaction costs between companies and other actors (search and information costs), bargaining and decision costs, and policing and enforcement costs (Maskell, 1999). Then, as Maskell (1999: 7) states: “Firms in communities with a large stock of social capital will always have a competitive advantage to the extent that social capital helps reduce malfeasance, induce reliable information to be volunteered, cause agreements to be honored, enable employees to share tacit information, and place negotiators on the same wave-length. This advantage gets even bigger when the process of globalization deepens the division of labor and thus augments the needs for coordination between and among firms.”

Previous literature has already pointed out the benefits of using external sources of information. For example, Tether (2000), analyzing the United Kingdom’s Innovation Survey, showed that firms that are more likely to rely on external sources of knowledge have introduced more novel innovations than others that did not. Romijn and Albu (2001), using the same database, show that small high-technology firms that are more likely to rely on external sources, such as science laboratories, universities, and financial institutions, have introduced major product and process innovations. In the same line, other studies have found that networking is beneficial in terms of survival, growth, and innovativeness (Hakansson and Snehota, 1989; Utterback, 1994; Gemünden et al., 1996; Littunen, 2000; Littunen and Virtanen, 2009).

In brief, innovation should be understood as a coherent process that should be properly designed based on the specific firm’s objectives and needs. The innovation process involves the action of a complex network (internal and external) that should interact and cooperate consistently and intensely in order to generate trust and reliable and effective communication that facilitates invention, as well as the successful implementation of an invention.

## A MacIntyrean account of innovation

Several authors (Bray, 2010; Martinez-Echeverría and Scalzo, 2015; Squires, 2021) have sought an anthropological view of innovation, explaining that neither the ancient world nor modernity have been able to interpret the true essence of innovation, and postmodernity is faced with the challenge of finding the right approach, as well as its connection with human nature.

As Martinez-Echeverría and Scalzo (2015) show, the ancient world rejected innovation, since the concept of the world that prevailed there was that of eternal circularity in which changes were attributed to the whim of Fortune. The emergence of cities propelled a shift from household economics—based on nature and aimed at mere subsistence—to a social division of labor with the aid of the market. Even though some philosophers—such as Plato—rejected the idea of wealth from anything other than nature –and so were reluctant to accept craftsmanship and commerce, Aristotle (1985 *Nicomachean Ethics*, henceforth NE, V; 1995 *Politics* I) foresaw the potential of human practical affairs and, in his own way, promoted innovation.

Indeed, human beings can innovate because they are constitutively open to gift and novelty, that is, they do not limit themselves to what has been given to them (as animals and machines do), but rather aspire to an end that exceeds their strength and individual attainment. This openness to “the beyond’ is the foundation of innovation; it implies having a conception of the good, as well as an ordered account of the associated means and ends. Innovation is a consequence of human freedom, classically understood as ‘the power to keep changing,” that is, to continually discover new possibilities and, as a result, open new horizons (Martinez-Echeverría and Scalzo, 2015: 8).

This approach remained for centuries and, even though inventiveness was notably present during the Middle Ages, innovation’s potential was not fully unleashed until modernity. Indeed, as the economist Keynes (1930: 359) noted, “[f]rom the earliest times of which we have record– back, say, to two thousand years before Christ– down to the beginning of the eighteenth century, there was no very great change in the standard of life of the average man living in the civilized centers of the earth.” However, development of the modern political-economic regime involved radical change when it comes to how human beings’ relationship with nature is understood. Guided by the idea of linear progress, a new view of human inventiveness’ potential for improving human life by adding novelty emerged.

Postmodern economics rejected the myth of indefinite progress, returning in some ways to a situation that parallels the ancient world by recognizing the ambivalent character of innovation. However, as a remnant of modernity, postmodernity has retained innovation’s shift from action itself to the resulting product; as a result, innovation’s end is now more associated with increased monetary gain rather than with value creation (*cf.* Drucker, 1986; Peters, 1997; Prahalad, 2009; Kotler and Amstrong, 2020). The current paradigm –guided by the maximization principle– supports a strange type of innovation that produces continuous multiplication of different products with no relation between them and no final sense of purpose. Furthermore, new technologies that attempt to replicate human intelligence and that perform certain technical tasks much faster and more accurately than humans seem to have taken the lead in the labor market (Raisch and Krakowski, 2021) where conversations about the possibility of the end of work lie in wait (Rifkin, 1995). In this context, the virtue ethics theory built on MacIntyre (2007) contributions may help to re-interpret the true meaning of innovation in connection with the practical character of human action and its contribution to the common good.

### Innovation as a (domain-relative) practice

In order to advance in that direction, we propose that innovation should be understood as a practice. MacIntyre defines practice as “any coherent and complex form of socially established cooperative human activity through which goods internal to that form of activity are realized in the course of trying to achieve those standards of excellence which are appropriate to, and partially definitive of, that form of activity, with the result that human powers to achieve excellence, and human conceptions of the ends and goods involved, are systematically extended” (MacIntyre, 2007[1981]: 175).

Examples of practices include chess, basketball, farming, or architecture. Practices are a “universal feature of human cultures” (MacIntyre, 1994: 287) and fundamentally imply two elements: “internal goods,” or goods that cannot be achieved outside cooperative activities, and the “standards of excellence” by which related performances are judged. Practices cultivate different faculties for excellence, as well as a suitable understanding of specific ends and goods internal to those activities; the connection to virtues is clear.

MacIntyrean practices seek goods internal to activities through the correct performance of the activities themselves. Practices are never individual and isolated activities, but always have a complex, communal, and social dimension. Internal goods can only be pursued through specific practices (they are “path-dependent”), and standards of excellence only make sense to previously initiated individuals. Practices demand cooperation and collaboration, not competition. One learns how to perform a practice by following experts. Experts are (potentially) unlimited, and they promote further excellence and development of the practice.

“[I] nstitutions are characteristically and necessarily concerned with … external goods. They are involved in acquiring money and other material goods; they are structured in terms of power and status, and they distribute money, power and status as rewards” (MacIntyre, 2007[1981]: 194). Institutions seek and deliver external goods (wealth, power, status) which can be attained through alternative ways. They incentivize zero-sum competition. All external, independent, impartial observers can recognize an institution’s standards of excellence, which are usually defined according to an objective, self-explanatory metric.

Institutions are important because they “sustain not only themselves, but also the practices of which they are the bearers. For no practices can survive for any length of time unsustained by institutions” (MacIntyre, 2007[1981]: 194). Institutions provide the external goods for the survival and development of practices. In this vein, Moore (2002) warns that although goods of effectiveness (external goods) should not be sought for themselves, neither should they be hypocritically disdained (Moore, 2002) because goods of excellence (internal goods) can only be achieved with their help. Thus, effectiveness is pursued to the extent that it leads to excellence (MacIntyre, 1988; Moore, 2005a); indeed, effectiveness and excellence could become mutually reinforcing (Moore, 2005a,b). Institutions and practices, with their respective external (effectiveness) and internal (excellence) goods, are intimately related, forming “a single causal order” (MacIntyre, 2007[1981]: 194).

How can innovation be characterized in these terms? Innovation may be understood as a practice, and more specifically, the type of practice that Beabout (2012) calls “domain-relative.”

Innovation is a complex activity that demands cooperation among experts. It is not the result of “individual geniuses”; rather, innovative practices are the result of collective forms of work. Innovation does not blossom in an individualistic and competitive culture; it requires collaboration among specialists.

Innovation requires resources (external goods such as money or specific material goods) that are provided by the institution that hosts the practice of innovation. Those institutions may be a corporation, university, or public research institute, depending where the specific field to which the innovation is referred takes place.

The standards of excellence of innovation may not be judged by an external observer as they are not based on objective evaluation criteria or calculable goals. Excellence in innovation is connected with the way (virtues) individuals involved in the innovation process carry out their work, the extent to which outcomes are connected to the strategic goals and *raison d’être* of the institution that host the practice, with the personal goals of individuals, and the degree to which innovation adds value to society, which implies a worldview about what is desirable in a specific context as conducive to flourishment.

At the same time, innovation requires domain-relative skills (innovation in car manufacturing requires different skills than innovation in pharmaceutical products or in education), not just multipurpose or general ones. Thus, innovation can be pictured as a practice related to a specific domain, with the practical activity housed by an institution (firms, universities, research institutes, public support organizations, etc.). It can be explained as a complex social and cooperative activity with standards of excellence known only to the practitioners.

### Creativity and practical wisdom as the excellences or virtues of innovation

The virtue that corresponds to the practice of innovation is creativity. In this paper, by defining creativity as a virtue we differentiate it from the *capacity* that most people have. It is understood rather as an excellence of character. In this vein, Kieran (2014: 125) explains that a person with the virtue of creativity “is someone who has acquired a certain degree of mastery and knows what she is doing in coming up with novel and worthwhile ideas or artifacts. In doing so, she is motivated by the values internal to the relevant domain and chooses what she does for reasons that hook up with those values in the right kind of ways.”

Astola et al. (2021) highlight three components of the virtue of creativity, namely its teleological, procedural, and motivational elements. The teleological part refers to “coming up with novel and worthwhile artifacts” and implies the successful reaching of the end-goal or *telos* of creativity. What “valuable” or “worthwhile” means should be determined in accordance with the nature of the activity undertaken in each specific context. The procedural component is associated with the idea that creativity as a virtue also requires “a certain degree of mastery” and that the agent “knows what she is doing” with her practice. This requires that creative persons have the kind of control over their creations that would separate them from random or unintended designs and constructions. The conception of mastery seems to imply authorship. Mastery requires a match between the artist’s intentionality and new, surprising, and valuable output, in such a way that the agent ultimately “knows what they are doing.” In MacIntyrean terms, working toward mastery of a practice involves engaging in the debates about the goods internal to the practice, in this case, innovation. Finally, the motivational element of creativity requires being motivated by the values inherent to innovation. The values required for intrinsic motivation can be the values internal to the sub-discipline of innovation that the innovator is working with. These three components of creativity are consistent with an interpretation of innovation as a domain-relative practice with creativity as the corresponding virtue.

Creativity is connected with other virtues such as perseverance, curiosity, reflexivity, and especially practical wisdom which exercises a directive and integrative function among virtues (NE, 1145a) following the “unity of the virtues” thesis. It has a central role in coordinating different moral virtues, ruling out conflicts. Practical wisdom is defined as the virtue of choosing the suitable means to the right end (NE 1144a). It implies doing the right thing, the right way, for the right purpose, and under the right circumstances (NE 1126b), and demands deliberation and decision-making (NE 1140a,b). Just as practical wisdom directs action toward the right end, it also provides a sound motivational element (Moberg, 2006).

Indeed, it can be seen that MacIntyrean practices resemble instances of Aristotelian practical wisdom (*cf.* MacIntyre, 1998; Sison and Redín, 2022). It is not difficult to see how these features of practical wisdom are connected with the teleological, procedural, and motivational components of creativity and how they are necessary for the practice of innovation.

### Innovation and tradition

Practices are embedded in individual biographies, which are, at the same time, rooted in sociocultural and historic communities. These communities comprise what MacIntyre calls “traditions.” Social and historical contexts or (following the MacIntyrean terminology) “communities of belief” have to provide the right ordering of rules, practices, virtues, and goods. This derives from their relation to the *arche* (first principle) and *telos* (final end) of practical life: human flourishing (*eudaimonia*).

Individuals inhabit a “community of belief” and develop virtues by engaging with its tradition (MacIntyre, 2007[1981]: 221–223). Part of individual identity is formed by being bearers of tradition: “the individual’s search for his or her good is generally and characteristically conducted within a context defined by those traditions of which the individual’s life is part, and this is true both of those goods which are internal to practices and of the goods of a single life” (MacIntyre, 2007[1981]: 222). Virtues are necessary to sustain practices, and also to keep traditions alive. Creativity and practical wisdom are never independent of tradition, which enables the foundation of communities through time.

MacIntyre (1988[1981]: 12) understands tradition as “an argument extended through time in which certain fundamental disagreements are defined and redefined in terms of two kinds of conflict”: external and internal. The former conflict arises among individuals that come from different traditions; the latter occurs among individuals within the same tradition. These conflicts primarily center on goods that provide traditions with a purpose (MacIntyre, 2007[1981]). Rational inquiry, development, and progress take place along with innovation as participants discriminate between what is superficially good and what is truly good, between what is good in a specific context and what is always good. This process of inquiry leads to the perfection of knowledge, when the *arche* and *telos* of practical life are fully integrated. Only then will it be possible “to deduce from it [*arche* or *telos*] every relevant truth concerning the subject matter of inquiry; and to explain the lower-order truths will precisely be to specify the deductive, causal and explanatory relationships which link them to the *arche*” (MacIntyre, 1988: 80).

“Narrative quest” is another expression for *arche* or *telos*-seeking rational inquiry (Moore, 2005a: 245–7; MacIntyre, 2016: 227–231); it implies that the unity of life is a plot, an enacted personal narrative in which each individual is subject and creator or co-creator of a unique story. Following MacIntyre (2007[1981]: p. 218) claim that a narrative makes human action distinctive and intelligible, Pinto-Garay et al. (2022a) agree that the intelligibility conferred upon an action depends much more on a particular agent’s narrative than on its relationship with practices (MacIntyre, 1988). Hence, personal narratives help individuals to understand how to contribute to a bigger purpose in terms of a common good; in other words, narratives give meaning and purpose to moral life. The eventuality and singularity of flourishing derive from the contingency and particularity of practical reason and action. Hence the necessity of dialog and joint search among communities: “the good life for man is the life spent in seeking for the good life for man” (MacIntyre, 2007[1981]: 219).

In MacIntyrean terms, innovation implies taking part in a tradition of inquiry regarding the principle and the end of practical life. Innovation is a cultural process of value creation. Individuals, by innovating, prudentially update the meaning of a tradition within their community. According to Martinez-Echeverría and Scalzo (2015), innovation does not need to destroy in order to create, as Schumpeter (1934) argued. Rather, within history, innovation develops processes, activities, and artifacts that did not exist before, making possible different ways of understanding the world and human life, as a result of free human action. History is a collaborative, free process that entails the continuous creation or destruction of value. Innovation does not occur in a vacuum, but rather within a community and a tradition, assuming the course of history and participating in it. Innovation, as a shared practice, implies a community’s shared vision of the world and is constitutive of a tradition; it adds value only when it contributes to the wellbeing of that community, i.e., the common good.

Traditions provide existential forms of coexistence that facilitate and improve human life. The ongoing accumulation of innovations, however large or small, carried out over the years by different communities and traditions have to be taken into account in a tradition, since they are precisely the changes that support a culture, with their respective visions of the world and man. However, it is possible for a tradition that at a certain moment served to facilitate coexistence to become useless, or even counterproductive, when the social circumstances that gave rise to it as an innovation have altered, making a further change necessary in order to improve human wellbeing.

In short, innovation, described as a domain-relative practice with creativity and practical wisdom as its corresponding virtues, can properly be understood within a certain tradition that historically and communally participates in inquiry regarding the common good as the final end of practical life.

## Artificial innovation and artificial creativity

In this section, we explore the impact of current AI advances on innovation in order to gauge whether it is possible (or realistic) to have AI-driven innovation processes. The Oxford Dictionary (2022) defines AI as “the theory and development of computer systems able to perform tasks normally requiring human intelligence, such as visual perception, speech recognition, decision-making, and translation between languages.” The field is principally organized around the goal of constructing a systems-based form of intelligence (Russell, 1997) based on six different sub-disciplines: natural language processing, knowledge representation, automated reasoning, machine learning, computer vision, and robotics (Russell and Norvig, 2016). The increasing computational complexity inherent in AI in general and these sub-disciplines in particular continues to generate extraordinary outcomes across a whole range of tasks previously thought to be impossible for software systems to handle (Luger, 2005). The key question for our study is whether artificial intelligence might be able to innovate on its own.

First, it is important to consider the scope of AI applications’ engagement in business processes, and thus their role in innovation. From the beginning, AI originated in the field of computer science, and its early commercial applications are found in relatively narrow domains like robotics. Nowadays, the learning algorithms that are being developed aim to become “general purpose technologies” and ultimately have applications across a very wide (supporters would say almost boundless) domain of application. Examples include machine learning algorithms like random forest, extreme gradient boosting, support vector machines, or deep learning.

The latter is one of machine learning algorithms’ most famous techniques and, in the last 10 years, it has outperformed the results of other algorithms. The mathematics of deep learning algorithms statically imitates the structure of neurons in the human brain. It uses a hierarchy of nonlinear transformations applied to input data to create output in the form of a statistical model. Iterations continue until the output has reached an acceptable level of accuracy. Related programs require access to immense amounts of training data and processing power (Ng, 2017).

The label “deep” refers to the number of processing layers through which data must pass. Deep learning models, as well as other machine learning techniques, require human supervision by AI experts. They have become state-of-the-art in several areas such as image recognition (face and object recognition from images or videos, which has been straightforwardly applied to self-driving cars), natural language processing (text classification, sentiment analysis, spoken language understanding, machine translation, writing style recognition), visual art processing (detecting the style period or imitating a certain style period to create new figures or synthesizing several styles), medical image analysis (cancer cell classification, organ segmentation or injury detection to help improve a diagnosis), and financial fraud detection among others.

There are four main limitations of machine learning models. The first is that they learn through observation, and they only know the data with which they have been trained. Data have to be sufficiently representative for output models to be generalizable. The second is that, once they have been trained, machine learning models become inflexible and cannot handle multitasking. They can provide efficient and accurate solutions but only to one specific problem and solving even a similar problem would require training the system again. Outputs lack robustness in dealing with novel situations. Third, machine learning algorithms cannot perform any task that requires reasoning or long-term planning, even with large data. Finally, they are currently programmed and supervised by human AI experts, who set a given problem’s optimization function and input variables, thus revealing a lack of self-sufficiency, a characteristic of human intelligence and creativity.

At the next level of technical ambition, we find Human-level AI (HLAI). There, AI is installed in “machines that think, that learn, and that create” (Herb Simon quoted in Russell and Norvig, 2016: 27). In spite of some authors’ (Good, 1966; Vinge, 1993; McCarthy, 2007) expectations, HLAI is not a reality at present. Machine learning and deep learning alone are not enough to produce human-level intelligence (Chollet, 2019). Scholars remain skeptical about their capabilities to replace human faculties, advocating more in favor of an “augmentation thesis” whereby AI technologies extend, enhance, and complement human skills (Epstein, 2015; Lemaignan et al., 2017; Jarrahi, 2018; Raisch and Krakowski, 2021).

The second aspect of AI’s participation in innovation considers its capacity to transform the process itself. Some AI applications just bring lower-cost or higher-quality inputs to many existing production processes. Yet, the latest advances, such as deep learning, aim to change the very nature of the innovation process while also guaranteeing profitability. In this context, Cockburn et al. (2019: 118) talk about machine learning as the “invention of a method of invention,” but Mitchell (2019: 146) hypothesizes that rather than being a general-purpose technology, AI may just be an “efficient method of imitation.” Machine Learning sees what “works” (by some criterion) and finds ways to exploit that relationship. (Mitchell, 2019). This is very useful for cost-saving and reducing complexity. For example, the development of personalized medicine has decreased the workload and complexity of cancer diagnosis. Litjens et al. (2016) show how deep learning techniques help improve the objectivity and efficiency of analysis, allowing pathologists to automatically identify, without human intervention, cancer, and micro and macro-metastases, while excluding 30–40% of cases with benign and normal tissue.

Third, we have to consider how technology operates and deals with uncertainty since it lies at the core of innovation and is the source of future opportunities and novelty (Knight, 1921). These are situations in which the future is unpredictable (Townsend and Hunt, 2019) and unbounded (Shackle, 1974), giving rise to infinite different possible world states. Following machine logic, “uncertainty consists in not knowing which state is the true one” (Arrow, 1974: 33), even though this is key to identifying the “correct manipulation of means” to achieve success in innovation through error reduction and adaptation in settings whose probabilities escape measurement (Knight, 1942). In recent years, several AI applications have been developed to help deal with uncertainty and to help researchers (augmentation thesis) to search through massive possibility sets to identify alternative opportunities (Agrawal et al., 2018). For example, in the chemical industry, deep generative models are used for inverse design (*cf.* Sanchez-Lengeling and Aspuru-Guzik, 2018), the objective of which is to discover tailored molecules from the starting point of a particular desired functionality. The input is the functionality and the output is the structure. Similar approaches have been applied in the fields of quantum physics and medical imaging.

Creativity is also related to exposure to and management of uncertainty. Boden (1998: 347–348) describes creativity as “a fundamental challenge for AI” and she supports the use of AI techniques to create new ideas in three ways: by producing novel combinations of familiar ideas (“combinational creativity”); by exploring the potential of conceptual spaces (“exploratory creativity”); and by transforming one or more dimensions of the space that enables the generation of previously impossible ideas (“transformational creativity”). Boden concedes that computer models are more successful at reproducing “exploratory creativity,” as it is difficult to approach the richness of human associative memory (necessary for simulating “combinational creativity”), and transformation requires advanced evaluation capacities that exceed current AI-models.

If the space is transformed then the resulting structures may not have any interest or value. Such ideas would be novel (to the AI-system, and in some cases to the entire previous history), but not necessarily creative (valuable, i.e., interesting, useful, beautiful, etc.). “This would not matter if the AI-system were able to realize the poor quality of the new constructions, and drop (or amend) the transformation accordingly” (Boden, 1998: 354). For example, Benjamin, the AI machine created by New York University, was used to create two fictional short films: *Sunspring* (2016) and *Zone Out* (2018). Regarding the former, Benjamin generated the film script based on an input of science fiction screenplays using Recurrent Neural Networks (RNN), which is a type of artificial architecture found in deep learning. For the latter (in less than 48 h), Benjamin collected public domain films and changed the voices, superimposed actors’ faces, and synchronized them with the background. Both cases resulted in random movies with unnatural scenes and story sequences. Benjamin was also used to create *It’s No Game* (2017), but in cooperation with humans. The outcome was a more natural and fluid film made in a more efficient way, yet the reviews of the movie are not particularly good.

Finally, one of the crucial underlying issues for assessing the (potential) capacity of AI to innovate corresponds to whether it might be able to perform a voluntary action, with the ability to choose and decide on its own actions rather than just follow an external principle. An “artificial” voluntary action may be expressed to the extent that the AI in use is autonomous. Verdicchio (2017) analyzes the different levels of autonomy applicable to AI (the repertoire of actions, the degree to which it may execute instructions outside the controlling program, whether the human programmer has already made “decisions,” the extent to which a machine can determine the course of action on the spot, and if a system is provided with the possibility of combining different actions within a simulation plan). He concludes that machines lack the necessary independent criteria at any of those stages. Following Barber and Martin’s (1999) understanding of autonomy, Verdicchio (2017) insists on AI’s inability to set its own goals. “Machines” (all kinds of computers and robots) are designed to perform specific tasks and functionalities with predetermined, externally-supplied goals. As he concludes, “Even the most advanced products of cognitive robotics and self-aware system research act on the basis of preprogrammed *desired behaviors* (the desire is clearly of the system designer’s; Verdicchio, 2017: 186).

In sum, in seeking to assess whether AI in its current state is able to carry out innovation processes on its own, we have seen how, at present, it has not reached the level of a genuine “general purpose technology.” Rather, its development has focused on specific domains that extend, enhance, and complement human skills (augmentation thesis); current AI is not a “method of invention” (Cockburn et al., 2019) *per se*, but rather a sophisticated method of imitation (Mitchell, 2019) that can recognize and identify patterns better and faster than the human mind. In that sense, AI is of great help in dealing with uncertainty, which is the natural environment for innovation, but it does not have the capacity to find creative solutions (useful, beyond novel) that are superior (or even comparable) to those that emerge from the human mind. So far, AI has managed to simulate certain capabilities found in the human mind, but it has not managed to acquire the autonomy necessary to carry out voluntary actions, nor has it proved capable of setting its own goals beyond those for which it has been programmed. As Anantrasirichai and Bull (2022: 637) state, *“*Humans will need to check the outputs from AI systems, make critical decisions, and feedback “faults” that will be used to adjust the model.”

## Is innovation specifically human?

At this moment in history, we are called on to rethink innovation and its role in history and in our daily lives. In view of the difficult economic and geopolitical situation over the last few years, many companies have emphasized innovation activities as a way to thrive and gain competitive advantage. Yet, in recent times, innovation has become detached from its aspiration to increase the existing value in society and has become progressively identified with monetary gain. Several authors (Bray, 2010; Martinez-Echeverría and Scalzo, 2015; Squires, 2021) have called for an anthropological view of innovation. We have attempted to fill this gap. Making use of MacIntyrean categories, we have presented innovation as a domain-relative practice with creativity and practical wisdom as its corresponding virtues. The virtue of creativity is understood as an excellence of character with three essential components that are teleological, procedural, and motivational. Practical wisdom involves doing the right thing, the right way, for the right purpose, and under the right circumstances, and demands deliberation and taking action. It exercises a directive and integrative function among virtues, and it is connected with the teleological, procedural, and motivational elements of creativity.

Finally, we have explained how innovation can only be understood within the course of history and a tradition. Innovation is a cultural process of value creation; in MacIntyrean terms, innovation requires taking part in a tradition of inquiry regarding the principle and end of practical life. Individuals, by innovating, prudentially update the meaning of tradition within the community.

Further, in recent years, we have witnessed an exacerbated process of robotization of business models. Increasingly, robots and AI applications co-exist with humans in the workplace. Several studies (Frey and Osborne, 2013, 2017; Bughin and Van Zeebroack, 2017; Hawksworth et al., 2018; Nedelkoska and Quintini, 2018) suggest that the advance of these technologies will significantly reduce the demand for human workers and the quality of work (Cherry, 2016). If we heed these predictions, there are important questions that arise that have not yet been addressed in the literature. For example, what would happen with innovation? Can machines and AI innovate?

In Section 4, we reviewed several issues related to machines’ capacity to lead innovation processes by themselves. We have explained that, at present, AI is not a genuine “general purpose technology” and its development has focused on specific domains that extend, enhance, and complement human skills (augmentation thesis); current AI is not a “method of invention” (Cockburn et al., 2019) *per se*, but rather a sophisticated method of imitation (Mitchell, 2019) that can recognize and identify patterns better and faster than the human mind. AI does not have the capacity to find creative solutions (useful, beyond novel) that are comparable (and far from superior) to those that emerge from the human mind. AI has not proved capable of setting its own goals beyond those for which it has been programmed and this prevents machines from carrying out voluntary actions.

At this point, we proceed to answer the question of whether machines or AI can innovate, or if this is a specifically human practice. We conclude that robots and “intelligent” devices do not have the capacity to innovate and they never will. They may replicate the human *capacity* of creativity, but they lack the necessary conditions to be a locus of virtue and engage within a tradition. Let us elaborate on several of these issues.

First, innovation requires cooperation among individuals; indeed, innovation is usually not attributed to an individual genius, but rather blossoms from the core of an innovation group (Hill, 2014). Cooperation implies a social dimension that robots do not have. Machines can coordinate, but not cooperate. Coordination is the mere orderly arrangement of powers to unite action toward the achievement of a common objective, whereas cooperation refers to collective efforts of persons voluntarily working in an initiative for the realization of a particular purpose (Consoli et al., 2006). Cooperation results from individuals’ willingness to help each other. Since AI and robots do not have free will (they just respond to pre-programmed instructions and/or optimize their response based on initial input, instructions, and parameters that are given) they are unable to voluntarily cooperate with peers, let alone to cope with uncertainty, something they are unable to manage (besides helping to reduce complexity).

Second, innovation requires pursuing internal goods to the practice, which implies developing virtues and forging character. Following an Aristotelian-Thomistic approach (as MacIntyre does), AI could not be considered an ethical subject (Sison and Redín, 2021): Neither AI nor robots exhibit the rational knowledge and free will necessary (in spite of attempts to endow machines with different degrees of autonomy and attribute to them some kind of aspirational “level of consciousness”) for voluntary action, or for virtuous action. Thus, innovation carried out by AI does not pursue the achievement of internal goods, and standards of excellence may be externally evaluated through any objective and quantitative criteria such as the number of patents registered or the derived economic returns. This implies that innovation can be motivated by the achievement of external goods (money, power, prestige) that lead to outcomes (processes, products, services) that do not necessarily add value to society and are not connected with the idea of *telos* or a final end.

Third, innovation only makes sense within a community and a tradition that seeks the best way to live. This implies freedom of action and a teleological orientation, whereas AI lacks free will and it cannot set its own goals. AI cannot function without rules, yet neither can it create its own. It depends on programmers. In addition, rules can only be written for a paradigm. For situations outside the paradigm, programmers will have to intervene and tweak the program. This does not happen with human beings who adapt to scenarios without external interventions. Machines cannot innovate because they are limited to what they have been given. Therefore, given the uncertain nature of the innovation process and its unexpected outputs throughout the entire process, machines are not able to constantly adapt to new circumstances.

Fourth, innovation involves prudentially updating knowledge. Innovation requires a perception of the context and existing needs. Limited to the data, it receives as inputs to optimize a reactive response in accordance with a given algorithm, AI fails again in this regard as perception *per se* goes beyond it. AI has no reasoning capacity and it cannot detect opportunities outside this paradigm. Knowledge demands at least some degree of consciousness and awareness (Botica, 2017), reflexivity, “knowing one knows,” an “I” or a “first-person perspective” (Baker, 2000: 91). Only by taking ownership over one’s thoughts and attitudes (Baker, 2000) may we participate in the practice of innovation. AI is also unable to see or experience truth, which consciousness requires. AI finds answers to programmed problems, and it can be sure that the answers are “correct,” which is different from being “true” (Penrose, 1991: 418). For AI, it is just a matter of “correct input,” “sufficient data” (Penrose, 1991: 419), and an appropriate algorithm with the corresponding amounts of training data and processing power.

Finally, AI cannot experience the “inspiration” (Penrose, 1991: 421) proper to scientific and artistic pursuits (Botica, 2017: 7), which consciousness enables humans to feel. This sort of “inspiration” is intrinsically linked to the capacity for creativity (it sparks combinational, exploratory, and transformational efforts to generate new ideas as a result of observation and reflection. As Henry Ford stated: “If I had asked people about what they wanted, they would have said faster horses”). Indeed, inspiration is one of the subjective emotions that can guide the virtue of creativity.

In short, we conclude that innovation is a (domain-relative) practice that is specifically human since it is strongly linked to virtue and can only be understood within the social embeddedness proper to communities and traditions. Innovation has a teleological component and implies prudential actions that demand rational knowledge and free will to act voluntarily. In its current state of development, AI does not exhibit these features.

Managers and economists should be conscious of the impact of automation to avoid a decline in innovation outputs and, in turn, in countries’ economic growth. If managers continue substituting human labor for machines, innovations will become merely incremental: for instance, they will allow companies to enhance the efficiency of existing processes, but they will never generate disruptive innovations that imply a totally new-to-the-world process. Thus, it is very important for companies to have a well-balanced innovation investment portfolio: companies should continue investing in incremental innovation to remain efficient and constantly enhance their products and services, but they must also invest in disruptive projects to remain competitive in the future. As the number of people in the innovation process is reduced and replaced by machines, disruptive innovations will decrease, slowing down the speed of development in countries and their economic growth.

Further research should study how AI could be integrated in the practice of innovation in a Human-Computer Interaction (HCI; Dix et al., 1993; Preece et al., 1994) process that does not denature the practice itself, but which would instead foster the development of internal goods along with techno-moral virtues (Vallor, 2016). Researchers and AI developers should seek to promote AI-systems as instruments that extend, enhance, and complement human skills (Epstein, 2015; Lemaignan et al., 2017; Jarrahi, 2018; Raisch and Krakowski, 2021) in a process of innovation that guarantees human responsibility and supervision at every point. This is a necessary condition for engaging AI-systems in innovation processes as it keeps intact the *raison d’être* of the overarching practice of innovation.

## Data availability statement

The original contributions presented in the study are included in the article/supplementary material, further inquiries can be directed to the corresponding author.

## Author contributions

DR contributed to the conception and design of the article, and wrote the first draft of the manuscript. GC-C and IR-C wrote subsequent versions of the paper. GS contributed to the revision of the paper in the review process. DR, GC-C, and IR-C wrote sections of the manuscript. All authors contributed to the article and approved the submitted version.

## Funding

Research Project PID 2021-124151NA-I00 funded by MCIN/AEI/10.13039/501100011033/ and by ERDF A way of making Europe.

## Conflict of interest

The authors declare that the research was conducted in the absence of any commercial or financial relationships that could be construed as a potential conflict of interest.

## Publisher’s note

All claims expressed in this article are solely those of the authors and do not necessarily represent those of their affiliated organizations, or those of the publisher, the editors and the reviewers. Any product that may be evaluated in this article, or claim that may be made by its manufacturer, is not guaranteed or endorsed by the publisher.
